# Association between menarche and iron deficiency in non-anemic young women

**DOI:** 10.1371/journal.pone.0177183

**Published:** 2017-05-09

**Authors:** Deepa L. Sekhar, Laura E. Murray-Kolb, Allen R. Kunselman, Carol S. Weisman, Ian M. Paul

**Affiliations:** 1 Department of Pediatrics, Penn State College of Medicine, Hershey, Pennsylvania, United States; 2 Department of Nutritional Sciences, The Pennsylvania State University, University Park, Pennsylvania, United States; 3 Department of Public Health Sciences, Penn State College of Medicine, Hershey, Pennsylvania, United States; 4 Department of Obstetrics and Gynecology, Penn State College of Medicine, Hershey, Pennsylvania, United States; Universidade de Sao Paulo, BRAZIL

## Abstract

**Background:**

The prevalence of iron deficiency (ID) among non-pregnant, reproductive-age US women significantly exceeds rates among males. In clinical practice ID screening relies on hemoglobin, a late-stage indicator of ID. As a single, low-cost laboratory test to diagnose ID before anemia develops is lacking, the study objective was to improve ID screening by identifying risk factors among non-anemic, iron-deficient reproductive age women.

**Methods:**

Cross-sectional data were from the National Health and Nutrition Examination Survey (NHANES) 2003–2010. Hemoglobin identified non-anemic women. ID was defined using the body iron formula, requiring ferritin and transferrin receptor values. Logistic regression assessed the association of sociodemographic, behavioral, and reproductive risk factors in an anemia-based conceptual framework with non-anemic reproductive age women (12–49 years) with ID, as well as subsets of younger (12–21 years) and older (22–49 years) women, recognizing that risks may differ by age.

**Results:**

Among 6216 women, 494 had ID (prevalence was 8.0%, 95% CI 7.3%, 8.6%). Among non-anemic younger women, 250 (8.7%, 95% CI 7.7%, 9.8%) had ID, compared to 244 (7.3%, 95% CI 6.4%, 8.2%) older women. Among younger women, menstruation for over 3 years was the only variable significantly associated with non-anemic ID (risk ratio 3.18, 95% CI 2.03, 4.96). No other significant risk factors were identified.

**Conclusion:**

Menstrual years was the only risk factor significantly associated with ID among non-anemic younger women. The negative results suggest ID risk factors among non-anemic women may need to be considered separately from those associated with ID anemia.

## Introduction

The prevalence of anemia and iron deficiency (ID) among non-pregnant, reproductive-age women in the United States far exceeds the prevalence among males [1, 2]. ID is perhaps the most common cause of anemia and certainly one of the easiest to treat when recognized [2–4]. As women deplete their iron stores, such as through blood loss from menstruation, anemia ultimately develops as a late-stage indicator of ID [3]. In clinical practice, ID screening is often based on a hemoglobin level to test for anemia due to its ease of interpretation and relatively low cost to obtain [3, 5]. Yet, this screening strategy misses a large number of “early stage” non-anemic, iron-deficient women.

Prevalence rates for ID among both anemic and non-anemic women are estimated at 9–16% [1]. Yet, the subset of non-anemic, iron-deficient women has been less well-studied. The symptoms of ID are insidious, so individuals tend to adapt to them over time, and they may be unlikely to voice concerns to their medical provider [3]. Non-anemic, iron-deficient women are known to have unrecognized symptoms including poor school performance, mood lability, and concentration difficulty which improve through iron supplementation [6–10]. Additionally, studies have suggested potential downstream negative effects of maternal ID for pregnancy and infant health [5, 11].

Multiple tests and combinations of tests have been proposed for diagnosing ID (e.g. ferritin, transferrin saturation, erythrocyte protoporphyrin, reticulocyte hemoglobin) [5, 12–17]. Unfortunately, all of these tests are either expensive, performed at limited laboratory facilities, or difficult to interpret, making them impractical for screening. From prior work, factors influencing ID *with anemia* among reproductive age women include race, poverty, education, low-iron intake, heavy menses, parity, contraceptive use, and tobacco use [5, 18–23]. Yet, little has been published on risk factors applicable to the subset of *non-anemic*, iron-deficient women.

Thus, the objective of this study was to utilize a conceptual framework of risk factors for ID anemia among reproductive age women (i.e. late-stage ID) to identify which risk factors might specifically apply to “early-stage” non-anemic, ID in non-pregnant reproductive age women using data from the National Health and Nutrition Examination Survey (NHANES) 2003–2010. As our prior work on ID anemia indicates risk factors differ between younger (12–21 years) and older (22–49 years) reproductive age women, we examined the group as a whole and subsequently by the above-mentioned age categories [24]. Identification of risk factors specific to non-anemic, iron-deficient women might be paired with current hemoglobin testing to improve early identification of the subset of women who require additional iron studies.

## Materials and methods

### Study participants

NHANES is a program of studies to assess the health and nutritional status of the US population [25]. Nationally representative surveys include both household interviews and physical examinations, which are conducted in mobile examination centers by the National Center for Health Statistics, Centers for Disease Control and Prevention (CDC). The NHANES interview questions encompass demographics, socioeconomics, diet, and health-related topics. Participants are selected via a stratified multistage probability with over-samplings of specific groups (e.g. African Americans, Hispanics) to generate reliable statistics [25].

We included all women 12 to 49 years of age with hemoglobin, ferritin, and soluble transferrin receptor laboratory values recorded in NHANES 2003–2010 to have an adequate sample size to consider risk factors for non-anemic ID. Inclusion and exclusion criteria were previously reported in a companion publication on ID anemia [24]. This project utilized a publically available, de-identified dataset and was approved by the Penn State College of Medicine Institutional Review Board.

### Conceptual framework and measures

A conceptual framework for the possible predicators of ID anemia was previously constructed based on a literature review, clinical expertise, and the consistency of selected variables measured in NHANES across the sample period [24]. In conducting this analysis the same framework was applied to considering risk factors for non-anemic ID.

#### Dependent variable

The dependent variable, non-anemic ID, was defined based on measures of hemoglobin (to separate out those women with anemia) and body iron (to determine ID), which was calculated from ferritin and soluble transferrin receptor. Methodology details regarding the assays and relevant normal ranges for hemoglobin, ferritin, and transferrin receptor have been previously reported [24]. The body iron formula developed by Cook et al. to determine iron status is as follows [1, 26]:

$$Body\;iron\;\left(\frac{mg}{kg}\right)=\;-[{\text{log}}^{10}\left(soluble\;transferrin\;receptor\left[\frac{mg}{L}\right]x\frac{1000}{ferritin\left[\frac{ug}{L}\right]}\right)-2.8229]/0.1207$$

The formula requires laboratory values for ferritin and soluble transferrin receptor and was used by NHANES to define the iron status of the US population starting in 2003. In this case a negative value (<0 mg/kg) is indicative of ID [1].

#### Independent variables

Details of the methodology regarding the independent variables in the model was also previously reported [24]. Independent variables included social determinants, behavioral determinants, and reproductive history [24]. Social determinants were as follows: 1) race/ethnicity categorized as Non-Hispanic White, Non-Hispanic Black, Mexican/Hispanic and other, 2) poverty defined using the family poverty income ratio, and 3) household food insecurity measured using the US Food Security Survey Module with responses dichotomized into full household food security versus marginal, low, or very low household food security. Behavioral determinants included: 1) tobacco or nicotine use within the last 5 days (yes/no), 2) iron intake using data from two NHANES dietary recalls, 3) elevated body mass index (BMI), and 4) physical activity assessed via a yes/no question [24]. Both BMI and physical activity were included as a proxy for poor nutrition, poor physical health, and low-iron intake. At both ends of the spectrum, e.g. frequent vigorous physical activity, low BMI and low physical activity, elevated BMI, there are associations with ID [27, 28]. Finally, reproductive history valuables included: 1) years since menarche and 2) ever using oral contraceptive pills or injectable contraception (e.g. medroxyprogesterone acetate). For older women only, time on oral contraceptives and number of live births were included. These data were not available for younger women. Additional details regarding the independent variables are available in our prior publication [24].

### Statistical analyses

Statistical analyses were performed using SUDAAN software, version 11.0.1, and in particular the RLOGIST procedure to fit logistic regression models (Research Triangle Institute, Research Triangle Park, NC) [29]. SUDAAN is a commercial software package designed for complex survey analysis. SUDAAN takes into account the sampling weights and complex study design to calculate proper variances of estimates.

Predictors were assessed for collinearity. For strongly correlated variables (e.g. age and years of menstruation or race/ethnicity and immigrant status), only one of the predictors was retained in the final model based on clinical applicability. Initially, bivariate analysis was used to examine the association of predictors with non-anemic ID. For ease of interpretation, potential predictors were converted to binary variables where possible.

Potential predictors of non-anemic ID were used to build a multiple logistic regression model. All predictors included in the conceptual framework were retained in the model regardless of significance level to gain a clear understanding of the impact of the various ID risk factors in non-anemic women. Reproductive age women were analyzed together and subsequently separated into younger (12–21 years) and older (22–49 years) age groups. Previous work demonstrates that younger and older women have different risk factors for ID anemia [24]. Thus, it was thought the same might apply in this analysis. Additionally, addressing age-specific risks may better inform medical providers for younger women (pediatricians, family practitioners) versus older women (internists, family practitioners, gynecologists). Descriptive data are reported as raw frequencies and percentages. From the multiple logistic regression analyses, model-adjusted risk ratios and corresponding 95% confidence intervals are reported [30]. Two models were constructed for older women, one with and one without the inclusion of measures unavailable for the younger women.

The method used in this analysis, following Bieler et al., provides appropriate model-adjusted risk ratios obtained directly from a logistic regression model in the NHANES complex sample survey setting [30]. These model-adjusted risk ratios from a logistic regression model are directly obtained as functions of the average marginal predictions [31, 32]. In a non-complex survey setting Bastos et al. have shown that the estimation of the adjusted risk ratio (i.e., prevalence ratio) from the log-binomial model, the Poisson model with robust variance estimation, and the logistic regression model using model-adjusted risk via marginal prediction (as presented here) all provide similar estimates [33]. Logistic regression with marginal prediction to estimate model-adjusted risk ratios was selected in this analysis with the following rationale: (1) to avoid issues of non-convergence for the log-binomial and Poisson regression with robust variance estimation, (b) to account for the various survey weights[31, 32], (c) as this method is implemented in the SUDAAN statistical software package, and (d) as the outcome is binary, logistic regression is appropriately bounded by 0 and 1, unlike the Poisson regression method of estimation.

## Results

### Characteristics of the sample population

A sample of 12,689 women aged 12 to 49 years participating in NHANES 2003–2010 was initially identified. This represented 18.9% of the NHANES 2003–2010 participants. After applying the exclusion criteria [24], the sample included 7,379 women, of which 6,602 had hemoglobin, ferritin, and transferrin receptor values in the dataset. Subsequently excluding the 386 women with anemia by hemoglobin left 6216 non-anemic women. Table 1 presents the characteristics of the sample population based on the predictors in the conceptual model. The prevalence of ID among the total sample was 494 (8.0%, 95% CI 7.3%, 8.6%). Among non-anemic younger women, 250 (8.7%, 95% CI 7.7%, 9.8%) were iron-deficient compared to 244 (7.3%, 95% CI 6.4%, 8.2%) older women.

**Table 1:** Characteristics of younger (12–21 years) and older (22–49 years) women.

Primary Outcome	All Women N = 6216^a^ n (%)	Younger N = 2869^a^ n (%)	Older N = 3347^a^ n (%)
Body Iron Deficiency			
Yes	494 (8.0)	250 (8.7)	244 (7.3)
No	5722 (92.0)	2619 (91.3)	3103 (92.7)
**Sample Characteristics**			
Race/Ethnicity			
White	2629 (42.3)	933 (32.5)	1696 (50.7)
Mexican/Hispanic	1818 (29.3)	962 (33.5)	856 (25.6)
Black	1471 (23.7)	830 (28.9)	641 (19.2)
Other	298 (4.8)	144 (5.0)	154 (4.6)
Poverty-income ratio			
<1.00	1500 (25.7)	868 (32.2)	632 (20.1)
≥1.00	4338 (74.3)	1824 (67.8)	2514 (79.9)
Food Security			
Full	4124 (67.3)	1791 (63.6)	2333 (70.3)
Not full	2008 (32.8)	1024 (36.4)	984 (29.7)
Tobacco/nicotine			
Yes	1038 (18.4)	285 (10.8)	753 (25.1)
No	4602 (81.6)	2359 (89.2)	2243 (74.9)
Recommended dietary allowance of iron			
0 days compliant	3010 (55.1)	1097 (42.8)	1913 (66.0)
1 day compliant	1489 (27.3)	769 (30.0)	720 (24.8)
2 days compliant	965 (17.7)	699 (27.3)	266 (9.2)
Body mass index			
Underweight	164 (2.7)	79 (2.8)	85 (2.6)
Normal	3060 (49.6)	1765 (63.4)	1295 (38.8)
Overweight	1524 (24.7)	519 (18.4)	1005 (30.1)
Obese	1417 (23.0)	466 (16.5)	951 (28.5)
Vigorous Physical activity			
Yes	2656 (43.3)	1634 (58.6)	1022 (30.5)
No	3479 (56.7)	1154 (41.4)	2325 (69.5)
Menstrual Years			
<3 years		858 (34.2)	
≥ 3 years		1654 (65.8)	
<25 years			1440 (48.4)
≥ 25 years			1538 (51.7)
OCPs^b^/Depo-Provera/Injectables			
Yes	3172 (57.6)	675 (26.8)	2497 (83.6)
No	2338 (42.4)	1848 (73.3)	490 (16.4)
Duration of OCP use			
0 years			597 (20.0)
<12 months			472 (15.8)
1–5 years			932 (31.3)
>5 years			978 (32.8)
Live Births			
never pregnant			613 (21.2)
0 or 1			590 (20.4)
2			857 (29.7)
>2			830 (28.7)

^a^Sample sizes listed are based on having a data recorded to calculate body iron deficiency. Sample sizes of the predictor variables may be smaller due to missing data.

^b^OCP(s), oral contraceptive(s)

### Multiple logistic regression

None of the variables in the conceptual model were significantly predictive of ID among non-anemic reproductive age women 12–49 years of age (Table 2).

**Table 2:** Multiple logistic regression model of body iron deficiency predictors for younger and older women combined (12–49 years), N = 4650.

Predictor	Risk Ratio (95% CI)	Overall P-value
Race		0.14
Mexican/Hispanic vs. White	1.28 (0.88, 1.87)	
Black vs. White	1.68 (1.08, 2.61)	
Other vs. White	1.43 (0.72, 2.84)	
Body mass index		0.72
Underweight vs. Normal	0.77 (0.30, 1.95)	
Overweight vs. Normal	1.21 (0.77, 1.91)	
Obese vs. Normal	0.94 (0.58, 1.53)	
Household Food Security		0.77
Marginal/Low/Very Low Food Security vs. Full Food Security	1.06 (0.69, 1.64)	
Family Poverty-to-Income Ratio		0.89
< 1.00 vs. ≥ 1.00	1.03 (0.65, 1.63)	
RDA^a^ Iron		0.19
Compliant with US RDA on both days vs. Not compliant with US RDA on either day	0.98 (0.59, 1.64)	
Compliant with US RDA on 1 day vs. Not compliant with US RDA on either day	0.66 (0.41, 1.06)	
Tobacco/Nicotine in last 5 days		0.20
Yes vs. No	0.61 (0.29, 1.30)	
Vigorous Physical Activity		0.45
Yes vs. No	0.83 (0.50, 1.36)	
Ever used OCPs^b^/Depo-Provera/Injectables		0.97
Yes vs. No	0.99 (0.68, 1.44)	

^a^RDA, recommended dietary allowance;

^b^OCPs, oral contraceptives

The final model of ID predictors in non-anemic young women is depicted in Table 3. Only menstruation for more than 3 years was significantly associated with ID (risk ratio 3.18, 95% CI 2.03, 4.96, p<0.001).

**Table 3:** Multiple logistic regression model of body iron deficiency predictors for younger women (12–21 years), N = 2095.

Predictor	Risk Ratio (95% CI)	Overall P-value
Race		0.37
Mexican/Hispanic vs. White	1.42 (0.83, 2.42)	
Black vs. White	1.56 (0.93, 2.62)	
Other vs. White	1.31 (0.59, 2.94)	
Body mass index		0.78
Underweight vs. Normal	0.93 (0.24, 3.55)	
Overweight vs. Normal	1.00 (0.53, 1.90)	
Obese vs. Normal	1.43 (0.70, 2.91)	
Household Food Security		0.85
Marginal/Low/Very Low Food Security vs. Full Food Security	1.05 (0.61, 1.82)	
Family Poverty-to-Income Ratio		0.70
< 1.00 vs. ≥ 1.00	0.92 (0.58, 1.44)	
RDA^a^ Iron		0.07
Compliant with US RDA on both days vs. Not compliant with US RDA on either day	1.18 (0.68, 2.06)	
Compliant with US RDA on 1 day vs. Not compliant with US RDA on either day	0.64 (0.40, 1.03)	
Tobacco/Nicotine in last 5 days		0.46
Yes vs. No	0.71 (0.28, 1.79)	
Vigorous Physical Activity		0.46
Yes vs. No	1.17 (0.77, 1.77)	
Ever used OCPs^b^/Depo-Provera/Injectables		0.93
Yes vs. No	0.97 (0.53, 1.79)	
Menstrual Years		<0.001
≥3 years vs. <3 years	3.18 (2.03, 4.96)	

^a^RDA, recommended dietary allowance;

^b^OCPs, oral contraceptives

The final model of ID predictors in older, reproductive-age women is depicted in Table 4. Again, none of the variables were significantly predictive of ID. The addition of live births and years of oral contraceptive pill use to the model for older, reproductive-age women did not result in any changes to the final model.

**Table 4:** Multiple logistic regression model of body iron deficiency predictors for older women (22–49 years), N = 2535.

Predictor	Risk Ratio (95% CI)	Overall P-value
Race		0.28
Mexican/Hispanic vs. White	1.20 (0.70, 2.05)	
Black vs. White	1.75 (0.97, 3.14)	
Other vs. White	1.59 (0.64, 3.92)	
Body mass index		0.48
Underweight vs. Normal	0.61 (0.17, 2.22)	
Overweight vs. Normal	1.24 (0.67, 2.28)	
Obese vs. Normal	0.80 (0.43, 1.48)	
Household Food Security		0.62
Marginal/Low/Very Low Food Security vs. Full Food Security	1.16 (0.64, 2.12)	
Family Poverty-to-Income Ratio		0.86
< 1.00 vs. ≥ 1.00	1.06 (0.54, 2.08)	
RDA^a^ Iron		0.44
Compliant with US RDA on both days vs Not compliant with US RDA on either day.	1.02 (0.45, 2.34)	
Compliant with US RDA on 1 day vs. Not compliant with US RDA on either day	0.67 (0.36, 1.27)	
Tobacco/Nicotine in last 5 days		0.17
Yes vs. No	0.55 (0.22, 1.33)	
Vigorous Physical Activity		0.34
Yes vs. No	0.71 (0.35, 1.45)	
Ever used OCPs^b^/Depo-Provera/Injectables		0.93
Yes vs. No	1.03 (0.56, 1.87)	
Menstrual Years		0.11
≥25 years vs. <25 years	1.44 (0.92, 2.24)	

^a^RDA, recommended dietary allowance;

^b^OCPs, oral contraceptives

## Discussion

Menstruation for greater than 3 years was the only risk factor for ID identified among a large, nationally representative sample of non-anemic younger reproductive-age women. No risk factors were significantly associated with ID among non-anemic older women or all non-anemic reproductive-age women. In the absence of a single, inexpensive screening test for iron-deficiency, current testing depends on the identification of anemia, a late stage indicator of ID, via a hemoglobin measurement [3, 5, 12]. Yet, it is concerning that the current anemia-based screening strategy misses a large subset of iron-deficient, non-anemic women who would benefit from treatment [5, 12].

These negative study results indicate that risk factors for ID without anemia may need to be considered separately of those for ID anemia. The lack of association may be secondary to a relative “lack of severity” in women who demonstrate ID without anemia, which is typically considered earlier stage than those who have depleted iron stores to the point of developing anemia [5]. For example, iron-deficient women may not report physical activity differently than women with normal iron stores as they are still able to compensate for their milder symptoms.

Rather than building a model based on risk factors for ID anemia as in this analysis, it may be preferable to target ID risk factor assessment to variables specifically associated with ID among non-anemic women. Halterman et al. reported on the association of lower standardized math scores among iron-deficient children and adolescents without anemia [6]. Murray-Kolb and Beard reported an improvement in measures of cognitive performance in non-anemic, iron-deficient women 18–35 years of age following iron supplementation [7]. Iron status in the absence of anemia has also been associated with central executive function in college women (Blanton et al.) [8]. Bruner et al. demonstrated improvements in verbal learning and memory when non-anemic, iron-deficient adolescent women were supplemented with iron for 8 weeks [9]. ID has also been linked to attention deficit hyperactivity disorder and restless leg syndrome [10, 34]. Thus, a successful ID risk assessment may need to move away from asking about risks such as diet and menses linked to ID anemia [18, 21], and instead include a brief cognitive assessment and questions on quality of sleep and concentration. Pairing such a risk assessment with the current hemoglobin test may significantly improve detection of non-anemic, ID.

Another explanation for the current findings may be varying definitions of ID [35]. NHANES adopted the body iron model used in the current analysis in 2003 [1]. Prior to this time the ferritin model had been used to assess iron status (this model identifies ID with 2 out of 3 iron laboratory parameters out of range) [1]. Additionally, publications have not adhered to a universal standard for measures such as hemoglobin and ferritin due to their wide variability based on age, sex, and race [35]. For example, a 1985 publication using NHANES data to assess the iron status of the US population based on the ferritin model and MCV model reported an association of impaired iron status and education and parity [36]. This association was not reproduced in this analysis.

### Limitations

The current study was limited by the available NHANES questions to consider as candidate risk factors for ID. The NHANES survey questions were not designed specifically for ID, but cover a broad range of health topics in a nationally representative sample of the U.S. population. In particular, the analysis would have been enhanced by the availability of questions related to menstrual blood flow that have been validated in reproductive age women. In addition, some measures available for older reproductive-age women (e.g., live births, detailed questions on duration and frequency of tobacco use) were not available in NHANES for adolescents.

## Conclusion

Among a nationally representative sample of non-anemic women, no risk factors for ID were identified except for menstruation for greater than 3 years among younger (12–21 years-old) reproductive age women. The negative results suggest iron deficiency risk factors among non-anemic women may need to be considered separately from those associated with iron deficiency anemia.
